# A ZO-1/α5β1-Integrin Complex Regulates Cytokinesis Downstream of PKCε in NCI-H460 Cells Plated on Fibronectin

**DOI:** 10.1371/journal.pone.0070696

**Published:** 2013-08-13

**Authors:** Saara Hämälistö, Jeroen Pouwels, Nicola de Franceschi, Markku Saari, Ylva Ivarsson, Pascale Zimmermann, Andreas Brech, Harald Stenmark, Johanna Ivaska

**Affiliations:** 1 Center for Biotechnology, University of Turku, Turku, Finland; 2 Medical Biotechnology, VTT Technical Research Center of Finland, Turku, Finland; 3 Department Human Genetics, K.U. Leuven, Leuven, Belgium; 4 Centre for Cancer Biomedicine, Faculty of Medicine, University of Oslo, Oslo, Norway; 5 Department of Biochemistry and Food Chemistry, University of Turku, Turku, Finland; University of Bergen, Norway

## Abstract

Recently, we demonstrated that integrin adhesion to the extracellular matrix at the cleavage furrow is essential for cytokinesis of adherent cells. Here, we report that tight junction protein ZO-1 (Zonula Occludens-1) is required for successful cytokinesis in NCI-H460 cells plated on fibronectin. This function of ZO-1 involves interaction with the cytoplasmic domain of α5-integrin to facilitate recruitment of active fibronectin-binding integrins to the base of the cleavage furrow. In the absence of ZO-1, or a functional ZO-1/α5β1-integrin complex, proper actin-dependent constriction between daughter cells is impaired and cells fail cytokinesis. Super-resolution microscopy reveals that in ZO-1 depleted cells the furrow becomes delocalized from the matrix. We also show that PKCε-dependent phosphorylation at Serine168 is required for ZO-1 localization to the furrow and successful cell division. Altogether, our results identify a novel regulatory pathway involving the interplay between ZO-1, α5-integrin and PKCε in the late stages of mammalian cell division.

## Introduction

Proper cell division is essential for health, since defects in chromosome segregation and cell division can lead to aneuploidy, which can promote tumorigenesis [1]. Cell adhesion to the surrounding matrix, mediated by integrins, governs tissue architecture and contributes to tissue homeostasis on several different levels. Adhesion dependent signaling supports cell cycle progression and survival [2]. In addition, integrins have emerged as important regulators of mitotic events [3]. Cell adhesion regulates cell shape and thus the orientation of the mitotic spindle and β1-integrins are important in spindle orientation in vitro and in vivo [4]–[7]. Survival and proliferation of normal adherent cells, like fibroblasts and epithelial cells, is critically dependent on cell adhesion. Upon detachment normal cells undergo a specialized form of cell death, anoikis [8] and under non-adherent conditions fibroblasts fail to execute normal cytokinesis [9], [10], demonstrating that adhesion is required for normal cell division.

Trafficking of integrins (via small GTPase Rab21) in the cell is important for cell migration and for successful cytokinesis [11]. During mitosis integrins traffic to the furrow to provide anchorage to the underlying matrix and facilitate RhoA activation at the ingressing furrow. Subsequently, the integrins are trafficked from the furrow to the opposing sides of the forming daughter cells to facilitate spreading in lamellipodia-like structures [11]. Interestingly, integrin traffic and cell migration are regulated also by protein kinase C epsilon (PKCε) [12], a kinase with an established role in cytokinesis [13], [14]. Thus, similar processes are employed by cells during migration and cell division.

We have demonstrated that lamellipodia stability and migration of interphase cells is supported by PKCε triggered formation of a complex of ZO-1 and α5β1-integrin on the plasma membrane [15]. Subsequently, these findings were confirmed by others and the pro-migratory role of ZO-1 in the lamellipodia was shown to involve the recruitment of MRCKβ, a Cdc42 effector kinase involved in the membrane protrusions [16]. Thus, ZO-1 plays an important role in integrin-mediated cell spreading but the requirement for ZO-1 in integrin-dependent cell division is not known.

In this study we demonstrate a role for a ZO-1/α5β1-integrin complex during cell division in NCI-H460 cells plated on fibronectin and reveal an unexpected role for tight-junction-protein ZO-1 in the regulation of integrins during cytokinesis. These data suggest a new level of co-ordination between cell-cell and cell-matrix adhesions in the proliferating epithelium.

## Materials and Methods

### Cell culture and DNA transfection

NCI-H460 non–small cell lung cancer cells were grown in RPMI 1640 medium supplemented with 10% fetal bovine serum (FBS), 1% Hepes buffer, 1% sodium pyruvate, 1% L-glutamine, and glucose (4500 mg/l; Sigma-Aldrich). Transfections were done with Lipofectamine 2000 (Invitrogen) or with HiPerfect Transfection Reagent (Qiagen) according to the manufacturer's protocol.

### Gene silencing and rescue

All gene silencing and rescue experiments were performed as described previously [15].

### Antibodies and reagents

The following antibodies were used in this study: anti-α5 (MCA1187, Serotec); anti-β1 (P5D2 (Developmental Studies Hybridoma Bank), anti-active β1 12G10 (Abcam) and MAB2252 (BD Transduction Laboratories)), anti–ZO-1 (mouse monoclonal antibody and rabbit polyclonal antibody, Zymed), anti-Plk1 (Abcam), anti-GFP (A11122, Molecular Probes), anti-GST (A5800, Invitrogen), anti-FLAG (M2, Sigma-Aldrich), SYBR Green I (Sigma-Aldrich), Anti-Mouse IgG - Mega 520 (Sigma-Aldrich) and α-tubulin (rat 6160-100, Abcam; mouse 12G10, Hybridoma bank). The PKCε antiserum was previously described [17]. Staining of filamentous actin was performed with phalloidin-488/561/647 conjugates (Invitrogen) or Atto 647N-Phalloidin (Sigma-Aldrich).

### Plasmids

The generation of siRNA-resistant α5-integrin expression constructs and WT, S168A and S168D ZO-1-Flag is described in [15]. The pEGFP-C1-ZO-1 construct is described in [18]. PH-PLC-GFP is described in [19] and was ordered from Addgene (Addgene plasmid 21179). The construct expressing GST-ZO1 PDZ1-3 was a kind gift from Dr. Walter Hunziker and GST-ZO1 PDZ2 has been described previously [20] followed by cloning into pGEX4T-1. The K253A mutation was introduced into siRNA-resistant pEGFP-C1-ZO-1, GST-ZO-1 PDZ1-3 and GST-ZO-1 PDZ2 using standard site directed mutagenesis (Qiagen).

### Immunofluorescence and microscopy

**Figure 1:**
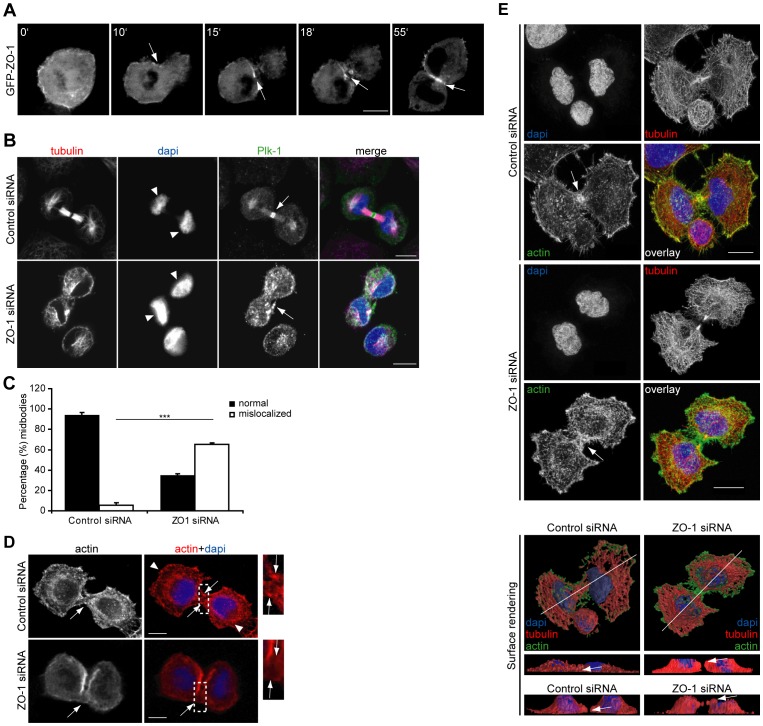
ZO-1 is required for cytokinesis in NCI-H460 cells on fibronectin. (A) Confocal sections depicting the localization of GFP-ZO-1 during cell division. Numbers indicate minutes and arrow indicates the furrow. (B, D) Control or ZO-1 silenced NCI-H460 cells in cytokinesis stained with antibodies against tubulin and Plk-1 (B) or phalloidin (D). DAPI was used to visualize nuclear morphology. Arrows point to midbodies and arrowheads to ruffles. (C) Quantification of midbody structures that show normal or abnormal localization in control or ZO-1 silenced NCI-H460 cells (n = 142 cells, ***, p<0.005). (E) STED super-resolution micrographs of ZO-1 or control silenced cells stained with Atto647-phalloidin and α-tubulin antibody (12G10) followed by Anti-Mouse IgG - Mega 520 together with a regular confocal channel to observe dapi stained nuclei. The lower panel shows surface rendered images of the cells shown in the top panels and additional side view examples of other representative cells. Cells in panels A, B, and D were on 5 µg/ml fibronectin, while cells in panel E were on uncoated precision cover glasses for high performance microscopes. Arrows point to midbodies. All scale bars are 10 µm.

Immunofluorescence and microscopy were done essentially as reported [15]. All cells analyzed by immunofluorescence (except for Stimulated Emission Depletion (STED) microscopy and the colocalization of α5β1-integrin with full-length GFP-ZO-1 WT and GFP-ZO-1 K253A) or time-lapse microscopy were plated in 10% FBS containing medium on fibronectin coated wells or coverslips, and allowed to adhere for 6–24 h before fixation with 3,7% PFAH or start of time lapse imaging. Cells for STED microscopy and for the colocalization of α5β1-integrin with full-length GFP-ZO-1 WT and GFP-ZO-1 K253A were plated on uncoated coverslips in 10% FBS containing medium. Fibronectin was coated by incubation with a 5 µg/ml fibronectin (from bovine plasma, Calbiochem) solution in PBS (1 h at 37°C), followed by blocking with a 0.1% BSA solution in PBS (1 h at 37°C). In PKC inhibition assays, calphostin C (2 µM) was added to NCI-H460 cells and incubated for 9 hours before further analysis. Immunofluorescent and time-lapse samples were imaged with a Zeiss Axiovert 200 M equipped with the spinning disc confocal unit Yokogawa CSU22 and a Zeiss Plan-NeoFluar 63× oil/1.4 NA objective (all immunofluorescence and time-lapse imaging shown in Figure 1A) or a Zeiss Plan-Neofluar 20× objective (all other time-lapse samples). Z-stacks were acquired with a step size of 0.25–0.75 µm and the maximum intensity projections were created using NIH ImageJ or Slidebook 4.2.0.7. software. Time-lapse imaging was performed under physiological conditions (37°C, 5% CO2). Movies from time-lapse experiments were created with NIH ImageJ software.

### Stimulated Emission Depletion (STED) microscopy

Control or ZO-1 silenced cells, grown on precision cover glasses for high performance microscopes (Marienfeld laboratory glassware), were fixed and stained using the same protocol as for immunofluorescence. Cells were stained for tubulin (1∶100 β-tubulin (12G10) followed by 1∶100 Anti-Mouse IgG - Mega 520 (Sigma-Aldrich)), filamentous actin (1∶50 Atto 647N-Phalloidin (Sigma-Aldrich)) and DNA (1∶4000 SYBR Green I (Sigma-Aldrich)). Images were acquired using a Leica TCS SP5 STED laser scanning microscope. Two sequentially scanned super resolution channels were used to image actin and tubulin. Nuclei were imaged with normal confocal channel. Acquired images were deconvoluted after background subtraction using Leica LAS AF software Version 2.6.07266 (Leica Microsystems), and then data sets were visualized using BioImageXD 1.0 software [21]. Surface rendering was used to create three-dimensional constructions. Surface rendering values, thresholds for multiple surfaces and other similar settings were set to images individually to obtain optimal visualization.

### Total internal reflection fluorescence microscopy (TIRF)

For visualization of substrate-adhering integrin, NCI-H460 cells adherent on glass-bottom live-cell dishes (MatTek) coated with 5 µg/ml fibronectin were incubated on ice with 1∶100 12G10 antibody for 30 minutes. Cells were fixed with 3.7% PFAH and incubated with Alexa-488 labeled secondary antibody and left in PBS. Samples were imaged with inverted wide-field TIRF microscope (Olympus CellR).

### Protein expression and purification

GST, GST-ZO1 PDZ1-3 (WT and K253A) and GST-ZO1 PDZ2 (WT and K253A) were produced and purified in *E. coli* strain Rosetta BL21DE3 according to the manufacturer's instructions (BD Biosciences).

### Enzyme-Linked ImmunoSorbent Assay (ELISA)

Four wells of a flat bottom, amine surface stripwell microplate (Costar) were coated per condition with 4 µM TAT-labeled WT or PPAA α5 peptide in 0.1 mM NaCO_3_ (pH 9.6) for 3 h at 4°C. The positively charged TAT-label was used (amino acids GRKKRRQRRRPQ) since it allows better binding of the peptide to the negatively charged amine surface. After blocking (2% BSA in TBST) for 2 h, 1 µg recombinant protein in blocking buffer (GST, GST-ZO1 PDZ1-3 (WT and K253A) or GST-ZO1 PDZ2 (WT and K253A)) was added and incubated for 1 h. Wells were then incubated 1 h with 30 ng rabbit anti-GST antibody in blocking buffer. Subsequently, wells were incubated with a horse radish peroxidase linked anti-rabbit antibody (GE Healthcare) for 1 h, stained using 1-step Ultra TMB ELISA (Thermo Scientific), and measured using an ENVISION 2100 multi-label plate reader (Perkin Elmer).

### Surface Plasmon Resonance (SPR)

SPR experiments were performed using a Biacore 2000 (GE Healthcare) as described previously [22]. GST-ZO1-PDZ1-3 (0.093 µM to 3 µM), preincubated with 10 µM PPATSDA α5-integrin peptide when indicated, were perfused over the immobilized lipids. Corrected equilibrium response units were plotted against the protein concentration using GraphPad Prism (GraphPad Software).

## Results

### ZO-1 is required for normal cytokinesis progression

To investigate the spatio-temporal localization of ZO-1 during mitosis, we imaged GFP-tagged ZO-1 in cells. GFP-ZO-1 accumulated to the area of constriction along the cell edges and appeared to concentrate in the furrow, finally localizing to the membranes between the daughter cells (Figure 1A, Video S1). This specific localization implied a potential role in cell division which was investigated with loss-of-function experiments in NCI-H460 cells. We chose these lung epithelial cancer cells due to their endogenous ZO-1 expression and the established requirement for ZO-1 and α5-integrin in their migration [15]. ZO-1 was silenced using a specific siRNA with no detectable off-target effects (Figure S1A; [15]) and cells were fixed and stained with tubulin and midbody marker Plk-1 antibodies. ZO-1 silenced cells displayed defects in cytokinesis (Figure 1B, C), characterized by delocalized Plk1 staining at the side of the daughter cells (Figure 1B, arrows) instead of the normal localization in-between the cells (Figure 1B). In addition, the symmetry of the cytokinesis was impaired such that most nuclei in ZO-1 silenced daughter cells showed a perpendicular orientation compared to the predominantly parallel orientation of the nuclei in control daughter cells (Figure 1B).

Given that ZO-1 binds directly to F-actin and to several regulators of cytoskeletal dynamics [23], we hypothesized that the observed aberrant midbody phenotype in ZO-1 silenced cells could be due to changes in actin organization. Actin filaments were clearly present in midbodies of control silenced cells (Figure 1D, arrows) but appeared to be largely absent from the diffuse structure connecting the daughter cell bodies in ZO-1 silenced cells (Figure 1D, arrows). In addition, cell spreading with characteristic actin polymerization at the opposing poles (Figure 1D, Control siRNA, arrowheads) was not observed in the ZO-1 silenced cells. Next we imaged tubulin and actin in ZO-1 and control silenced cells using a Stimulated Emission Depletion (STED) microscope, which can improve the spatial resolution up to 50 nm (Figure 1E). From the high-resolution images it is evident that the formation of the actin contractile ring is impaired in the furrow of ZO-1 silenced cells (Figure 1E, arrows). In addition, in the ZO-1 depleted cells the furrow becomes disconnected from the matrix (Figure 1E, surface rendering, arrows). These results imply that during cytokinesis ZO-1 directly or indirectly affects the organization of the cytoskeleton, adhesion of the furrow and spreading of the daughter cells.

### ZO-1/α5β1-integrin complex is required for integrin mediated adhesion during cytokinesis

**Figure 2:**
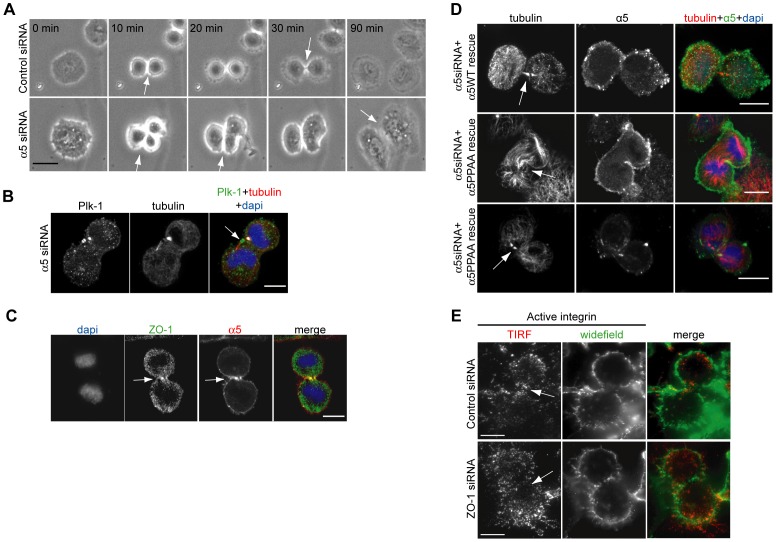
ZO-1- α5-integrin complex formation is required for cytokinesis. (A) Time lapse imaging (1 frame/10 min for 4 h) of control and α5-integrin silenced NCI-H460 cells. (B) Micrograph of α5-integrin silenced NCI-H460 cells stained as indicated. (C) NCI-H460 cell in cytokinesis stained as indicated. (D) α5-integrin silenced NCI-H460 cells expressing siRNA resistant WT or PPAA (unable to bind ZO-1) α5-integrin in cytokinesis stained as indicated. All cells were plated on fibronectin and dapi was used to visualize nuclear morphology. (E) TIRF (red) and epifluorescence (green) microscopy of ZO-1 or control silenced NCI-H460 cells stained for active β1-integrin during cytokinesis. All cells were growing on 5 µg/ml fibronectin. All arrows indicate the cleavage furrow. All scale bars are 10 µm.

Integrins regulate cytokinesis and inhibition of integrin-mediated adhesion in the furrow induces aberrant cell divisions with a multinuclear phenotype [11], [24]. Integrin α5 is the main fibronectin receptor expressed in NCI-H460 cells [15]. To address the requirement for α5β1-integrin in the division of these cells, we followed α5-integrin silenced cells plated on fibronectin using time-lapse microscopy. Depletion of α5-integrin (Figure S1B) resulted in impaired cell divisions with tripolar or bipolar furrowing and finally daughter cells fusing together in telophase (Figure 2A, Videos S2, S3) whereas control silenced cells divided normally. Importantly, integrin silencing resulted in an abnormally positioned midbody (Figure 2B, arrow) and non-parallel nuclei in the daughter cells (Figure 2B), similar to ZO-1 depleted cells (Figure 1B), confirming that α5-integrin is needed for cytokinesis and indicating that ZO-1 might regulate integrins during cytokinesis in these cells.

Since integrins are recruited to the cleavage furrow [11], we wanted to investigate whether endogenous ZO-1 and α5-integrin localize to the furrow upon cell adhesion to fibronectin. ZO-1 and α5 were both detected at the furrow (Figure 2C, arrows) indicating a possible co-operative function during cell division. Integrin α5 interacts with ZO-1 via its cytoplasmic tail C-terminus, in which two sequential proline residues (residues 1092 and 1093) are critical for complex formation and ZO-1 dependent cell migration [15]. To study whether ZO-1 regulates adhesion of α5-integrin also during cell division, we expressed siRNA-resistant α5-integrin WT and PPAA (ZO-1 binding mutant) constructs in α5-integrin silenced cells plated on fibronectin and investigated cell division (Figure 2D). In cells expressing the α5WT construct, cell division and midbody formation was normal, as indicated by integrin staining along the membrane and furrow and with the tubulin antibody, respectively (Figure 2D, upper panel, arrow). Cells expressing the α5PPAA mutant, however, showed cytokinesis defects with either asymmetrical constriction or, similar to ZO-1 silenced cells, misaligned midbody structures (Figure 2D, two lower panels, arrows). These results suggest that α5-integrin binding to ZO-1 is required to support division of NCI-H460 cells and interestingly, the STED imaging implies that ZO-1 depletion disrupts the physical contact of the cleavage furrow to the matrix.

This was studied further using total internal reflection fluorescence (TIRF) microscopy (Figure 2E). Active, ligand binding competent β1-integrins can be detected specifically with a conformation-dependent monoclonal antibody (12G10, [25]). Imaging the localization of active β1-integrins within 100 nm of the substrate (TIRF) or through-out the cell (widefield) showed that in control silenced cells, active β1-integrin is recruited to the cleavage furrow, where it localizes to the underlying fibronectin matrix (Figure 2E, arrows, ‘TIRF’). However, in ZO-1 siRNA cells some active β1-integrin was detected in the midzone (Figure 2E, arrowheads, ‘widefield’) but not in the proximity of the matrix (Figure 2E, arrows, ‘TIRF’). These results correlate well with the disengaged furrow detected with STED and imply that ZO-1 is required for appropriate α5β1-integrin mediated adhesion during cell division.

### ZO-1 PDZ2 binding to α5-integrin and PI(4,5)P2 are mutually exclusive

**Figure 3:**
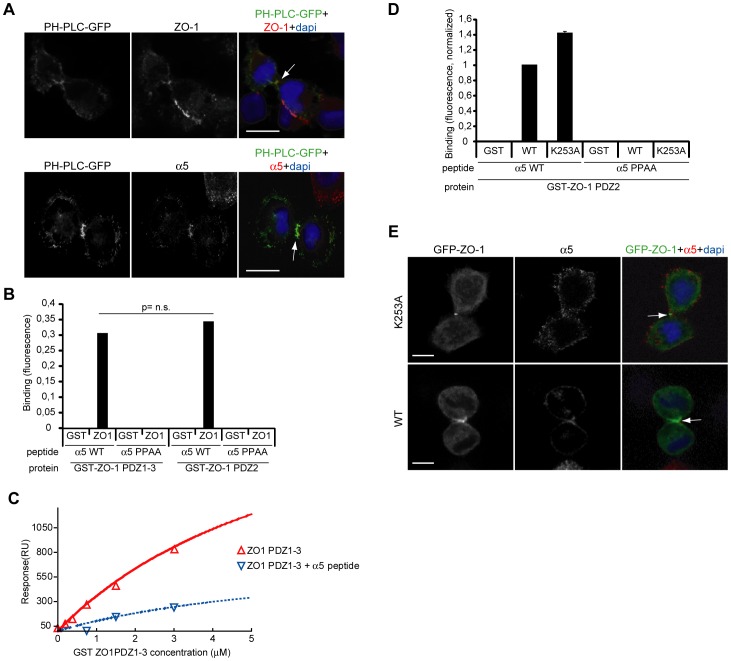
ZO-1 PDZ2 binds PI(4,5)P2 and α5-integrin. (A) NCI-H460 cells in cytokinesis expressing low level of PI(4,5)P2 binding PH-PLCδ-GFP and stained as indicated. Arrows indicate furrow. (B, D) ELISA assay for interaction between recombinant WT (B, D) or K253A (D) GST-ZO-1 PDZ1-3 or PDZ2 domains and α5-integrin peptides: α5 WT (PPATSDA) and α5 PPAA (AAATSDA). (C) Surface plasmon resonance measurements of interaction between GST-ZO-1-PDZ1-3, with (blue dotted line) or without pre-incubation (red line) with α5 WT peptide, and PI(4,5)P2. Binding of GST-ZO-1-PDZ1-3 to PI(4,5)P2 was corrected for non-specific binding. (E) NCI-H460 cells in cytokinesis expressing GFP-ZO-1-WT or lipid-binding deficient GFP-ZO-1K253A and stained for α5-integrin. Cells in panel A were growing on fibronectin coated coverslips, while cells in panel E were on uncoated coverslips. Arrows indicate the furrow. All scale bars are 10 µm.

Phosphoinositides play important roles in recruiting proteins to membranes and in the regulation of the cytoskeleton. Phosphatidylinositol 4,5-bisphosphate (PIP2) is mainly localized at the plasma membrane and accumulates at the furrow during cytokinesis where it is required for the plasma membrane-actin linkage during cell division [26]. The subcellular localization of PIP2 can be imaged in live cells with low level expression of the PH-domain of PLCδ fused to GFP [26]. Using this probe we observed that ZO-1 and α5β1-integrin both colocalized with PIP2 in dividing NCI-H460 cells on fibronectin (Figure 3A). Integrin α5 interacts with the N-terminal PDZ domain-containing (PDZ domains 1–3) part of ZO-1 in motile cells [15]. Using an enzyme-linked immunosorbent assay (ELISA) with GST-ZO-1 PDZ1-3 and PDZ2 domains we now showed that the PDZ2-domain was sufficient to bind the α5-integrin tail (α5 WT; c-terminal residues PPATSDA, Figure 3B). Importantly, mutation of the α5 tail double-proline motif, which is essential for ZO-1 binding [15], abolished this interaction. These data define the PDZ2-domain as the α5-integrin binding site and, as the ZO-1 PDZ2 domain can bind PIP2 [20] and binding of certain proteins, like the gap junction protein connexin43, to the PDZ2 domain of ZO-1 and ZO-2 has been shown to be mutually exclusive with lipid binding [20], the relationship between integrin and PIP2 binding to ZO-1 was investigated. We performed a surface plasmon resonance (SPR) experiment measuring interaction between ZO-1 PDZ domains and PIP2. In agreement with previous studies [20], ZO-1 PDZ1-3 fragment interacted with PIP2 in a concentration dependent manner. Interestingly, pre-incubation of ZO-1 with α5 peptide strongly reduced binding to PIP2 (Figure 3C), suggesting that PIP2 and integrin binding to ZO-1 are mutually exclusive.

The ZO-1 PIP2 interaction requires the K253 residue in the PDZ peptide binding groove [20]. However, mutation of this residue into an alanine did not inhibit α5 WT peptide binding to ZO-1 (GST-ZO-1-K253A PDZ2 fragments) in the ELISA assay (Figure 3D), suggesting that despite their ability to compete for ZO-1 binding in the SPR assay, α5-integrin and PIP2 interact with distinct residues within ZO-1-PDZ2. In line with this, we observed similar colocalization of α5β1-integrin with full-length GFP-ZO-1 WT and GFP-ZO-1 K253A in the cleavage furrow (Figure 3E, arrows), suggesting that PIP2 binding is not required for ZO-1 localization to the furrow during cytokinesis. However, it is important to note that ZO-1 is capable of forming stable homodimers [27] and thus we cannot rule out the possibility that endogenous wild-type ZO-1 may facilitate the recruitment of the PIP2 binding deficient ZO-1 to the furrow. Taken together, these findings suggest a molecular basis for two independent ZO-1 interactions during cell division: recruitment and positive regulation of α5β1-integrin in the adhering base of the furrow and PIP2 binding which could provide the necessary link between membrane bound PIP2 and the actin ring [26].

### PKCε expression and phosphorylation of ZO-1 affect cytokinesis in NCI-H460 cells on fibronectin

**Figure 4:**
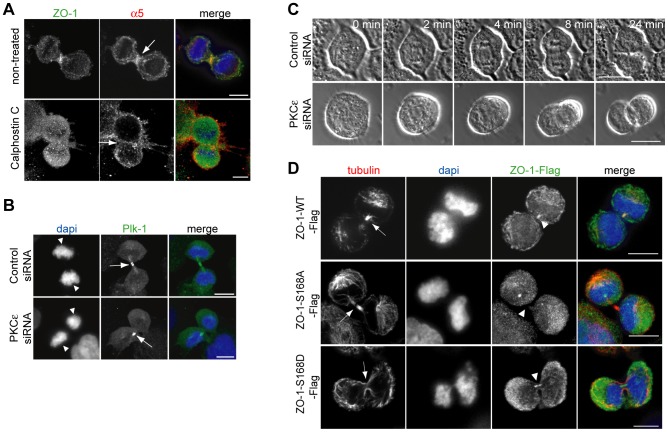
PKCε and ZO-1 phosphorylation are necessary for cytokinesis. (A) Immunostaining of NCI-H460 cells treated with or without 2 µM PKC inhibitor Calphostin C stained as indicated. (B) Immunostaining of control or PKCε silenced NCI-H460 cells in cytokinesis stained as indicated. (C) Time-lapse imaging of control and PKCε silenced NCI-H460 cells (1 frame/min for 30 minutes). (D) Immunostaining of NCI-H460 cells expressing WT or phosphomutants S168A or S168D ZO-1-Flag in cytokinesis stained as indicated. DAPI was used to visualize nuclear morphology. All cells were growing on 5 µg/ml fibronectin. Arrows indicate the furrow. Arrowheads indicate nuclei (B) or FLAG-ZO-1 localization (D). All scale bars are 10 µm.

PKCs have been implicated in the regulation of ZO-1 subcellular distribution [15], as well as the intracellular traffic of integrins [28]. Interestingly, we found that 2 µM PKC inhibitor Calphostin C inhibited furrowing and the localization of ZO-1 and α5-integrin to the cleavage furrow when compared to the control treated cells (Figure 4A, arrows indicate the cleavage furrow). PKCε is required for cell division, particularly during the late stages of cytokinesis [13]. Therefore, to further investigate the possible requirement for PKCε specifically and to rule out non-specific effects of Calphostin C, we investigated whether PKCε regulates NCI-H460 cell division upstream of ZO-1 using siRNA-mediated PKCε depletion. The cytokinesis phenotype of PKCε depleted cells (Figure S1C) stained with dapi and a Plk-1 antibody (Figure 4B, arrows point to the midbody structures) closely resembled that in ZO-1 silenced cells (Figure 1B), including the non-parallel nuclei in the daughter cells (Figure 4B, arrowheads). Time-lapse imaging confirmed the essential role of PKCε for proper cell division. While control silenced cells separated into two daughter cells in less than 30 minutes, PKCε silenced cells frequently underwent aberrant separation (Figure 4C, Videos S4, S5) resulting in only one daughter cell due to cell fusion or cell death. This is in agreement with the reported occasional collapse back of the daughter cells in dividing PKCε null cells and indicates that PKCε may play a complex role in multiple different stages of mitosis [14]. We have shown earlier that the subcellular localization of ZO-1 is regulated, at least partly, by PKCε-dependent phosphorylation of serine 168 in ZO-1 [15]. To test the requirement for this site during cytokinesis, cells were transfected with Flag-tagged WT, S168A or S168D ZO-1 constructs and stained for tubulin, Flag and dapi. Cells expressing ZO-1-WT-Flag underwent normal cell division with ZO-1- and tubulin-rich midbodies (Figure 4D, arrowheads and arrows). In contrast, telophase cells expressing ZO-1-S168A-Flag exhibited abnormal midbody structures (Figure 4D, arrowheads and arrows), resembling those in cells lacking ZO-1 (Figure 1B). In cells expressing ZO-1-S168D-Flag, on the other hand, ZO-1 localization and midbody formation (Figure 4D, arrowheads and arrows) was similar to that in WT ZO-1-Flag expressing cells. These data show that during cell division, PKCs regulate recruitment of ZO-1 and α5-integrin to the furrow and implicate PKCε-dependent phosphorylation of ZO-1 at S168 in this process.

## Discussion

**Figure 5:**
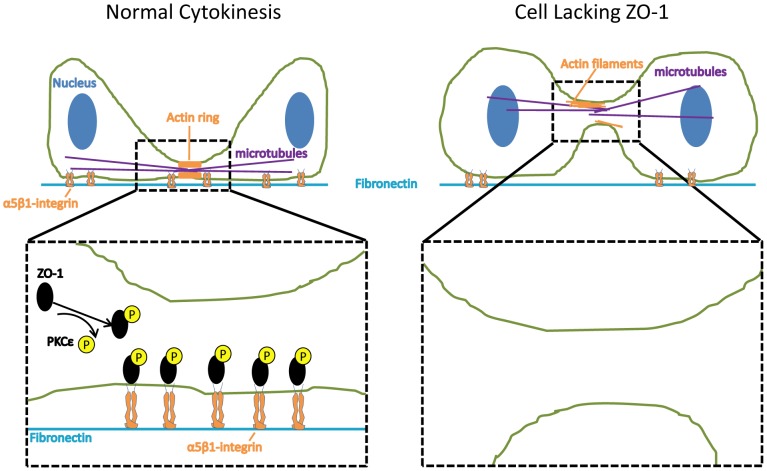
Model for the role of ZO-1, α5-integrin and PKCε in cytokinesis of epithelial NCI-H460 cells on fibronectin. During normal cytokinesis (left panel), PKCε-dependent phosphorylation of ZO-1 is required for interaction between α5-integrin and ZO-1. This interaction allows α5-integrin to mediate adhesion to fibronectin in the cleavage furrow, and this adhesion is required for successful cytokinesis. In addition, the ZO-1/α5-integrin complex contributes to actin assembly in the furrow. Cells that lack ZO-1 (right panel) fail to accumulate α5-integrin in the furrow which correlates with impaired cytokinesis. In addition, actin structures in the cleavage furrow of cells lacking ZO-1 are disorganized.

Here we have identified a novel pathway containing ZO-1, α5-integrin and PKCε with a role in cytokinesis of epithelial NCI-H460 cancer cell line on fibronectin (the model in Figure 5 summarizes our main conclusions). We show that α5-integrin mediated adhesion in the furrow is under the positive regulation of ZO-1, which in complex with α5-integrin contributes to correct actin assembly in the furrow. Unexpectedly, depletion of ZO-1 from cells results in loss of symmetry between the forming daughter cells and detachment and mislocalization of the midbody. Furthermore, mutagenesis of a PKCε-dependent phosphorylation site in ZO-1 impairs cytokinesis and the localization of α5-integrin and ZO-1 in the cleavage furrow. Thus, even though an exact molecular mechanism is still lacking, our data integrate several important biological observations into a common pathway.

The observations that ZO-1 deficient embryos can be viable till E11.5 [29] and that epithelial cells lacking ZO-1 seem to have no major mitotic phenotype [30] seem to contradict a role for ZO-1 in cytokinesis. However, ZO-1 interacts exclusively with α5-integrin [15] and therefore ZO-1 is only expected to play a role in cytokinesis when cells depend on α5-integrin for adhesion, which under the aforementioned conditions is unlikely as levels of this integrin are low in epithelial cells. However, α5-integrin levels are high in some cancer types [31], [32] and its overexpression has been associated with increased malignancy and metastasis and decreased survival [33], which opens the possibility that ZO-1 plays a role in cytokinesis in certain transformed cells.

At present the specific function of ZO-1 in the furrow remains unclear. Although we cannot rule out the possibility that the cytokinesis defects in ZO-1 depleted cells are due to defects in cell spreading during or after cytokinesis, this seems unlikely given the specific localization of ZO-1 in the cleavage furrow (Figure 1A) and the observation that α5-integrin mediated adhesion of the cleavage furrow depends on ZO-1 (Figure 1E and 2E). In tight junctions ZO-1 functions as a scaffold protein, connecting the transmembrane tight junction proteins to cytoplasmic proteins [23]. In addition, ZO-1 binds directly to F-actin and to several regulators of cytoskeletal dynamics [23]. We observed that in ZO-1 silenced cells actin is less polymerized in the furrow and the formation of the contractile ring appeared to be impaired. Since deregulated actin polymerization could generate uneven forces between cells and affect tubulin dynamics through actin-tubulin cross-talk [34], the loss of the ZO-1/actin link could explain the observed asymmetrical cytokinesis. Since myosin-II recruitment to intercellular junctions is inhibited upon ZO-1 depletion [35] and myosin-II motor activity is latent until it interacts with actin at the cleavage furrow [36], ZO-1 could also regulate myosin-II motor activity during cytokinesis. All these putative functions of ZO-1 in cytokinesis could be due to the scaffolding properties of ZO-1, which play a role in intercellular integrity [37] and promoting cell invasion [38].

The classic view of tight junctions (and ZO-1) is that in epithelial cells they stay intact at the plasma membrane at all stages of mitosis, including cytokinesis, merely to establish intercellular adhesions in the daughter cells [39]. However, the data provided here identify a conceptually new level of integration between cell-matrix and cell-cell adhesion regulators in cytokinesis and a pathway that is likely to be important for epithelial integrity and tissue homeostasis.

## Supporting Information

Figure S1 **Western blot analysis of NCI-H460 cells transfected with the indicated siRNAs and blotted for ZO-1, PKCε and α5-integrin as indicated.**
(TIF)

Video S1 **Time lapse imaging of GFP-ZO-1 localization during cell division in NCI-H460 cells.** Time stamp is h∶min∶sec.
(MOV)

Video S2 **Time lapse imaging of cell division in α5-integrin silenced NCI-H460 cells.** Time stamp is h∶min.
(MOV)

Video S3 **Time lapse imaging of cell division in control silenced NCI-H460 cells.** Time stamp is h∶min.
(MOV)

Video S4 **Time lapse imaging of cell division in PKCε silenced NCI-H460 cells.** Time stamp is h∶min.
(MOV)

Video S5 **Time lapse imaging of cell division in control silenced NCI-H460 cells.** Time stamp is h∶min.
(MOV)
