# Supplementary figures and images for: A ZO-1/α5β1-Integrin Complex Regulates Cytokinesis Downstream of PKCε in NCI-H460 Cells Plated on Fibronectin
Source: PLoS One. 2013 Aug 13;8(8):e70696. doi: 10.1371/journal.pone.0070696 (PMC3742740; doi:10.1371/journal.pone.0070696)

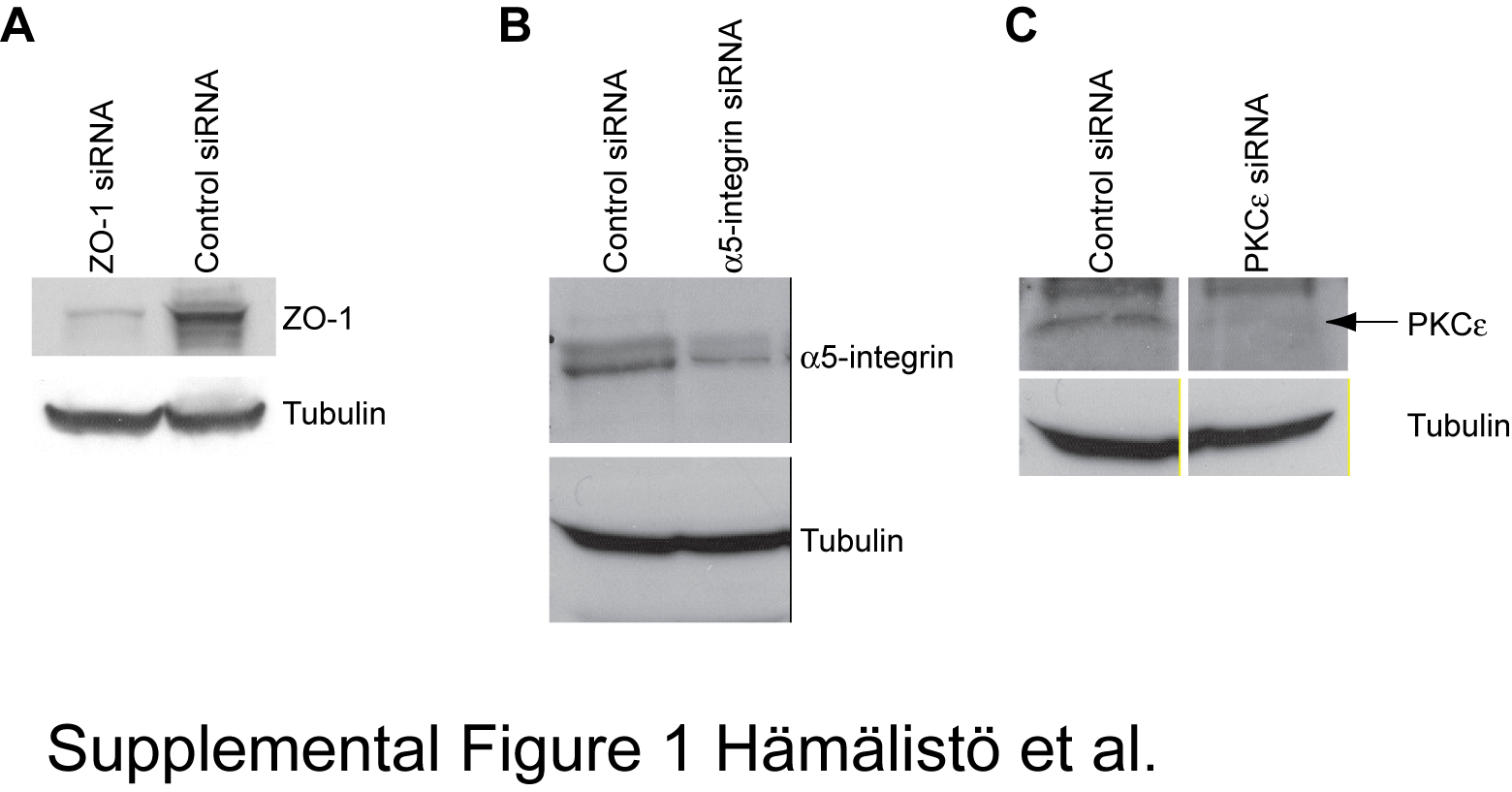

Supplement: Figure S1 — Western blot analysis of NCI-H460 cells transfected with the indicated siRNAs and blotted for ZO-1, PKCε and α5-integrin as indicated. (TIF) [file pone.0070696.s001.tif]
